# Comment on: “Prevalence and Influencing Factors of Malnutrition in Diabetic Patients: A Systematic Review and Meta‐Analysis”

**DOI:** 10.1111/1753-0407.70067

**Published:** 2025-03-03

**Authors:** Shubham Kumar, Nosaibah Razaqi, Rachana Mehta, Ranjana Sah

**Affiliations:** ^1^ Center for Global Health Research, Saveetha Medical College and Hospital, Saveetha Institute of Medical and Technical Sciences Saveetha University Chennai India; ^2^ Afghanistan Center for Epidemiological Studies Herat Afghanistan; ^3^ Scientific Research Committee Afghanistan Medical Students Association Herat Afghanistan; ^4^ Clinical Microbiology, RDC Manav Rachna International Institute of Research and Studies Faridabad India; ^5^ Department of Paediatrics Dr. D. Y. Patil Medical College Hospital and Research Centre, Dr. D. Y. Patil Vidyapeeth (Deemed‐to‐be‐University), Pimpri Pune India; ^6^ Department of Public Health Dentistry Dr. D. Y. Patil Medical College Hospital and Research Centre, Dr. D. Y. Patil Vidyapeeth (Deemed‐to‐be‐University), Pimpri Pune India

Dear Editor,

We read with great interest the recent article by Zhang et al., titled “Prevalence and influencing factors of malnutrition in diabetic patients: A systematic review and meta‐analysis” [1]. The study provides valuable insights into an important area of clinical nutrition. The authors should be commended for their effort in consolidating data on malnutrition in diabetic patients and highlighting its associated risk factors. However, upon a detailed review of the article, several methodological issues and potential areas for improvement were identified, which could enhance the reliability and clinical applicability of their findings.

One significant limitation lies in the presence of substantial heterogeneity across the included studies, as evidenced by high *I* ^2^ values (> 90%). The heterogeneity raises concerns regarding the comparability of pooled prevalence estimates for malnutrition and at‐risk malnutrition, which the authors reported as 33% and 44%, respectively. Although the authors performed subgroup analyses by measurement tools, region, and diabetes complications, these analyses did not fully address the underlying causes of variability. The authors could have considered using meta‐regression analysis to explore potential sources of heterogeneity, such as differences in study design, sample characteristics, and diagnostic criteria [2]. This statistical approach would have provided a deeper understanding of the heterogeneity and potentially improved the robustness of their conclusions.

Additionally, the authors relied on confidence intervals (CIs) to present pooled estimates but did not include prediction intervals (PIs). While CIs describe the precision of the pooled effect size, PIs would have conveyed the range of effects expected in future studies. The use of PIs is especially critical in the presence of high heterogeneity, as it offers a clearer picture of the variability across different settings and populations [3]. The inclusion of PIs alongside CIs would have strengthened the interpretation of the meta‐analysis results, particularly for clinical decision‐making.

Another important methodological concern involves the assessment of publication bias. The authors used Egger's test and visual inspection of funnel plots to evaluate publication bias. While these methods are widely used, they may not be optimal for meta‐analyses involving proportions, where asymmetry in funnel plots can arise from true heterogeneity rather than bias. The authors might have instead employed more appropriate approaches, such as the Doi plot and LFK index, which are specifically designed to assess publication bias in proportion meta‐analyses [4]. These methods offer greater reliability in detecting bias in prevalence studies and could have provided additional assurance regarding the integrity of the findings.

The use of diverse diagnostic tools, such as the Mini Nutritional Assessment (MNA), Nutritional Risk Screening 2002 (NRS‐2002), and Global Leadership Initiative on Malnutrition (GLIM), complicates result interpretation due to varying criteria and cut‐off values, contributing to heterogeneity. While acknowledged, the authors could have stratified their analysis by individual tools rather than pooling data indiscriminately. Advocating for a standardized malnutrition assessment tool specific to diabetic patients would improve consistency and comparability.

The analysis of influencing factors was limited by small sample sizes for certain variables, such as smoking, education level, and diabetic foot infections, reducing reliability. Future meta‐analyses should incorporate more studies or pool related data to enhance statistical power. Additionally, potential confounders, including socioeconomic status, dietary patterns, and psychological factors, were insufficiently addressed, despite their known influence on malnutrition risk in diabetics.

While the study by Zhang et al. is an important contribution to the field, addressing these concerns would provide greater clarity on this critical issue. The authors' efforts in this domain are appreciated, and we hope these suggestions will guide further advancements in the study of malnutrition in diabetic patients.

## Author Contributions

S.K., R.M., R.S., and N.R. critically provided comments on methodological aspects. S.K., N.R., and R.S. have written and edited the draft.

## Ethics Statement

The authors have nothing to report.

## Conflicts of Interest

The authors declare no conflicts of interest.
